# A hybrid seasonal prediction model for tuberculosis incidence in China

**DOI:** 10.1186/1472-6947-13-56

**Published:** 2013-05-02

**Authors:** Shiyi Cao, Feng Wang, Wilson Tam, Lap Ah Tse, Jean Hee Kim, Junan Liu, Zuxun Lu

**Affiliations:** 1 School of Public Health, Tongji Medical College, Huazhong University of Science and Technology, Wuhan, 430030, P.R., China; 2 JC School of Public Health and Primary Care, The Chinese University of Hong Kong, Hong Kong, SAR, China

**Keywords:** Hybrid model, Incidence, Prediction, Seasonality, Tuberculosis

## Abstract

**Background:**

Tuberculosis (TB) is a serious public health issue in developing countries. Early prediction of TB epidemic is very important for its control and intervention. We aimed to develop an appropriate model for predicting TB epidemics and analyze its seasonality in China.

**Methods:**

Data of monthly TB incidence cases from January 2005 to December 2011 were obtained from the Ministry of Health, China. A seasonal autoregressive integrated moving average (SARIMA) model and a hybrid model which combined the SARIMA model and a generalized regression neural network model were used to fit the data from 2005 to 2010. Simulation performance parameters of mean square error (MSE), mean absolute error (MAE) and mean absolute percentage error (MAPE) were used to compare the goodness-of-fit between these two models. Data from 2011 TB incidence data was used to validate the chosen model.

**Results:**

Although both two models could reasonably forecast the incidence of TB, the hybrid model demonstrated better goodness-of-fit than the SARIMA model. For the hybrid model, the MSE, MAE and MAPE were 38969150, 3406.593 and 0.030, respectively. For the SARIMA model, the corresponding figures were 161835310, 8781.971 and 0.076, respectively. The seasonal trend of TB incidence is predicted to have lower monthly incidence in January and February and higher incidence from March to June.

**Conclusions:**

The hybrid model showed better TB incidence forecasting than the SARIMA model. There is an obvious seasonal trend of TB incidence in China that differed from other countries.

## Background

Tuberculosis (TB) is an often fatal infectious disease caused by the agent *mycobacterium tuberculosis*. Despite concerted worldwide efforts to control this disease, TB remains a major health issue with a high global health burden, particularly in low and middle-income countries [1,2]. In 2009, it was estimated that there were approximately 9.4 million newly diagnosed cases, 14 million prevalent cases, and 1.7 million deaths that were attributable to TB in the world [3].

Since tuberculosis can be disseminated widely from active cases through aerosol droplets, it is highly cost-effective to detect a TB epidemic in its early stages in order to optimize disease control and intervention. One important characteristic of TB that assists in predicting epidemics is seasonality. A recent systematic review which reviewed studies from multiple regions around the world, noted a seasonal pattern of tuberculosis across countries. Moreover, the review noted that TB cases tended to reach their peak during the spring and summer seasons [4]. The seasonality of TB epidemics allows more efficient and effective use of existing data and it also provides clues to explore the environmental and social factors which might influence TB epidemics.

There were some retrospective studies using routine surveillance data to describe the patterns of TB occurrence [5,6], and the TB forecasting models have been developed in various countries to understand and predict the trajectory of a TB epidemic [7-9]. Up to the present, there has no such studies conducted in China, even though China possesses the world’s second largest tuberculosis epidemic after India.

Given the high priority to tuberculosis control, there have been large numbers of international TB prevalence studies. Nonetheless, measurements of prevalence were usually confined to the adult population and these surveys typically will not include culture-negative pulmonary TB cases [10]. Although there are increasing numbers of countries that possess TB prevalence data from representative, population-based surveys, there has been no nationwide survey of TB incidence in any country.

In order to address these noted gaps in the literature, in this study we aim to develop a model to predict TB epidemics and to analyze its seasonality in China. There are many models which can be used in infectious disease forecasting, such as Markov chain models [8], Grey models, general regression models, autoregressive integrated moving average class models (ARIMA) [11] and artificial neural network [12]. For better forecasting performance, hybrid models which combined two or more single models for communicable disease forecasting have also been explored, and previous findings indicate that hybrid models outperformed single models [13,14]. We plan to compare our model with a hybrid model in order to compare their performance. The findings of this study will be useful for forecasting TB epidemics and providing reference information for TB control and intervention.

## Methods

### Study area and data collection

The Ministry of Health of the People’s Republic of China (MOH) releases the incidence data of notifiable diseases on a monthly basis. The data including incidence and mortality rates of each notifiable disease is primarily collected from every local Center of Disease and Control. In the MOH, more than 30 of infectious diseases are categorized into three major types (ClassA ,B or C) of notifiable diseases. Tuberculosis is categorized to class B notifiable disease in this system. All cases must be verified by the clinical and laboratory diagnosis. All tuberculosis cases must be reported within twelve hours by health professionals to their local health department which will, in turn, be reported to the MOH. We collected all the monthly incidence data of TB from MOH from January 2005 to December 2011 (Table 1). Ethical approval is not required for this study.

**Table 1:** Reported monthly incidence number of tuberculosis from January 2005 to December 2011

**Month**	**Year**						
	**2005**	**2006**	**2007**	**2008**	**2009**	**2010**	**2011**
January	85577	89436	115457	111688	90160	105877	99617
February	75625	103823	91235	101689	127167	88759	98157
March	151935	147996	141508	156679	139986	138574	135848
April	165419	145029	148930	153978	139915	133833	129351
May	150823	135933	135933	142612	128411	128598	125129
June	151330	134879	134775	131699	142182	127545	119344
July	133807	123829	134695	136378	129537	122602	112647
August	132729	127509	130404	122923	126893	117221	115140
September	123062	115540	119078	123998	124152	112288	106925
October	104851	108177	111325	119821	109343	101463	100392
November	117468	111384	116503	110896	104782	110414	110662
December	116859	114262	119421	121114	120341	105036	104710

### Model development

**Figure 1:**
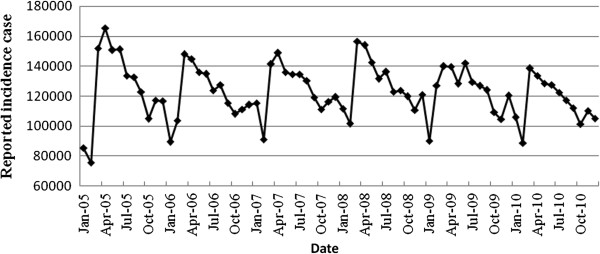
Reported monthly incidence number of tuberculosis from January 2005 to December 2010.

This study was based on time series data of TB. Before the model fitting, a line plot was drawn to observe the trend of the time series data for model selection (Figure 1). Based on this plot, we found that the time series data of TB incidence had a seasonality trend with periodicity. Firstly, we considered the ARIMA model, which have been widely used to analyze the time series data. Furthermore, artificial neural network was a model which was broadly applied in multivariate nonlinear analysis recently [14] and could be a supplement of linear analysis. Therefore, we selected seasonal ARIMA (SARIMA) model and hybrid model of SARIMA and generalized regression neural network (GRNN).

#### SARIMA model construction

The SARIMA model is developed from the ARIMA model. The future value of a variable in ARIMA model is a linear function of several past observations and random errors. It uses autoregressive parameters, moving average parameters and the number of differencing passes to describe a series in which a pattern is repeated over time. There are six main parameters for fitting the SARIMA model: the order of autoregressive (p) and seasonal autoregressive (P), the order of integration (d) and seasonal integration (D), and the order of moving average (q) and seasonal moving average (Q). In this study, SARIMA model was developed using monthly incidence of TB as the dependent variable and its past variables as independent variable with the control of seasonality. The general SARIMA model has the following form:

$$\phi \left(B\right)\Phi \left(B^{S}\right){\left(1-B^{S}\right)}^{D}X_{t}=\mathrm{\theta B}\Theta \left(B^{S}\right)\epsilon_{t}$$

with

$$\phi \left(B\right)=1-\phi_{1}B-\phi_{2}-\ldots -\phi_{P}B^{P}\Phi$$

$$\left(B^{S}\right)=1-\Phi_{1}B^{s}-\Phi_{2}B^{2s}-\ldots -\Phi_{P}B^{\mathrm{Ps}}$$

$$\theta \left(B\right)=1-\theta_{1}B-\theta_{1}B^{2}-\ldots -\theta_{q}B^{q}$$

$$\Theta \left(B^{S}\right)=1-\Theta_{1}B^{s}-\Theta_{2}B^{2s}-\ldots -\Theta_{Q}B^{\mathrm{Qs}}$$

In the equation, B is the backward shift operator, ϵ_t_ is the estimated residual at time *t* with zero mean and constant variance and X_t_ denotes the observed value at time *t* (*t* = 1, 2… k). The process is called SARIMA (p, q, d) (P, D, Q)^s^(*s* is the length of the seasonal period). Autocorrelation function and partial autocorrelation functions were performed to identify the six main parameters preliminarily. Akaike information criterion and Schwarz Bayesian criterion were used to determine the optimal model that most closely fit the data. The SARIMA model was built using a SPSS 13.0 (SPSS Inc., Chicago, IL, USA) and *p* value < 0.05 was used as a cut-off for statistical significance.

#### Development of the hybrid model combining SARIMA model and Generalized Regression Neural Network (GRNN) model

Artificial neural network models are being applied widely in multivariate nonlinear models. There are self-organizing and self-learning processes in it [15]. Neural networks are trained by a set of data with the outcomes that the trainer wishes the network to learn. Then the trained network can be evaluated by inputting similar but previously unseen data. Among various artificial neural network models, GRNN model is a universal approximator for smooth functions based on nonlinear regression theory. Given enough data, it is able to solve any smoothing function approximation problem. The training series of a GRNN consists of input values *x*, each with a corresponding value of an output *y*. estimated value of *y* can be produced minimizing the squared error. For constructing a better GRNN model, selection of an appropriate smoothing factor is very important. The smoothing factor plays a great role in matching its predictions to the data in the training patterns. The selection process is usually conducted by software.

Because the SARIMA model had been used to analyze the linear part of the actual data, the residuals should contain nonlinear relationships. In SARIMA- GRNN model, we combined the linear and nonlinear parts of the models. We selected the monthly estimated incidence number of TB at time variable *t* from the SARIMA model and time variable *t* as two input variables. There is one output variable *y* which was the actual reported monthly incidence number of TB. The iterations of GRNN learning and simulating data was conducted in Matlab 7.0 software package (Math Works Inc., Natick, MA, USA) to determine the relationship between the input and output variables.

### Comparison between the two models in simulation performance

Three indexes were used in comparisons of the fitting effectiveness of the SARIMA model and the hybrid model: the mean square error (MSE), mean absolute error (MAE) and mean absolute percentage error (MAPE). Their calculation formulas are:

$$\mathrm{MSE}=\frac{1}{n}\overset{n}{\underset{t=1}{\sum }}{\left(X_{t}-{\hat{X}}_{t}\right)}^{2}$$

$$\mathrm{MAE}=\frac{1}{n}\overset{n}{\underset{t=1}{\sum }}\left|X_{t}-{\hat{X}}_{t}\right|$$

$$\mathrm{MAPE}=\frac{1}{n}\overset{n}{\underset{t=1}{\sum }}\frac{\left|X_{t}-{\hat{X}}_{t}\right|}{X_{t}}$$

where X_t_ is the real incidence number at time *t*, ${\hat{X}}_{t}$ is the estimated incidence at time *t*, and *n* is the number of predictions. Good fitness performance is demonstrated with these three indices showing as small a value as possible.

## Results

**Figure 2:**
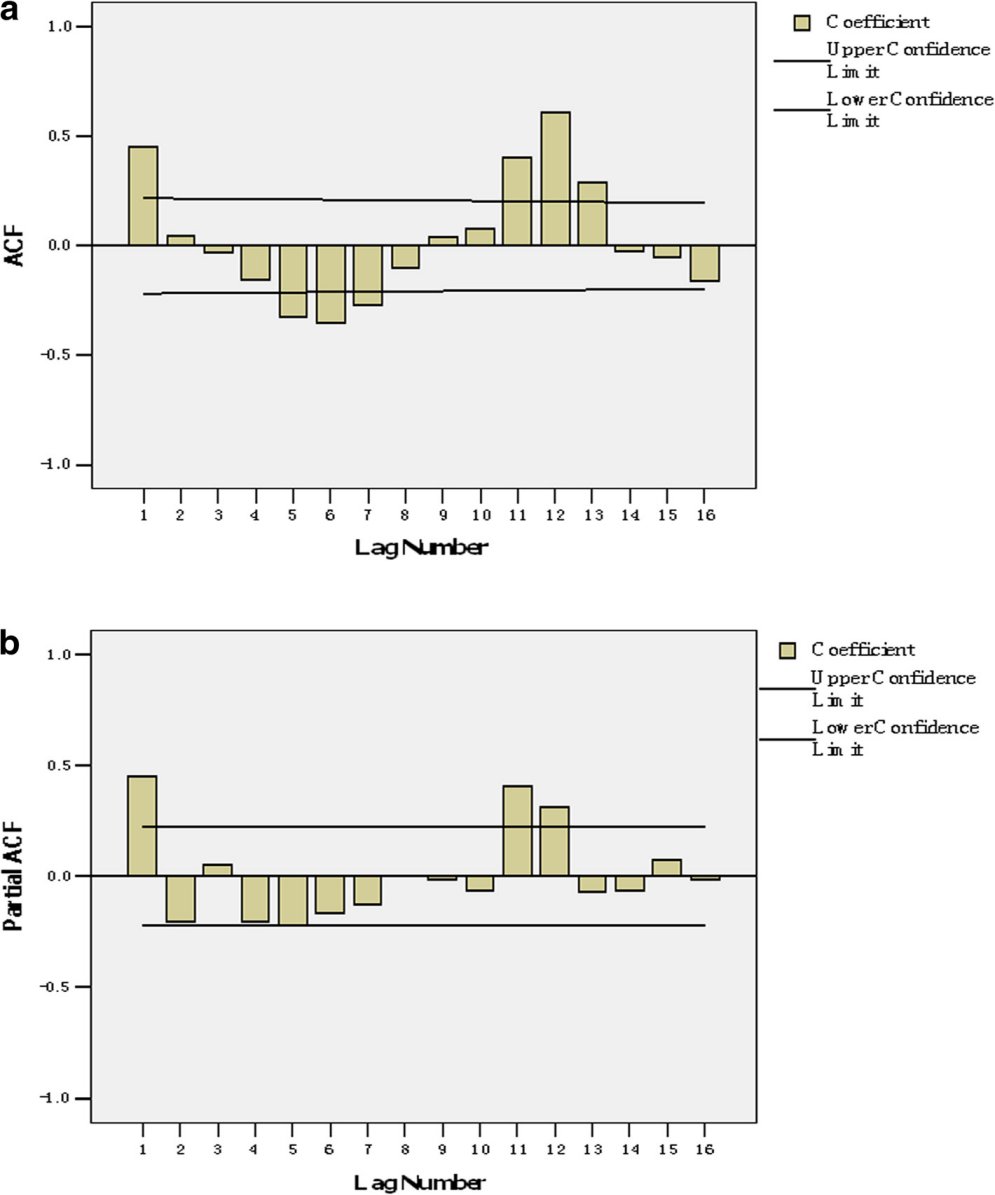
**Autocorrelation function(ACF) and partial ACF charts of monthly tuberculosis incidence numbers.** (**a**) ACF chart; (**b**) Partial ACF chart.

The data set from January 2005 to December 2010 was used to model fitting. For determining main parameters (p, P, d, D, q, Q) of SARIMA, we drew autocorrelation function plot and partial autocorrelation function plot (Figure 2). In the SARIMA time series analysis, the best model generated from the data set was SARIMA (1, 0, 0) (1, 0, 1)_12_. The model equation is: (1 − 0.272B)(1 − 0.997B^12^)X_t_ = (1 − 0.888B^12^) × 123088.518. The estimation of model parameters and their testing results were presented in Table 2.

**Table 2:** Parameter estimates and their testing results of the SARIMA model

**Coefficients**		**Estimates**	**Standard error**	***t***	***p *value**
Non-Seasonal Lags	AR1	0.272	0.097	2.793	0.007
Seasonal Lags	Seasonal AR1	0.997	0.020	49.893	0.000
	Seasonal MA1	0.888	0.392	2.268	0.026
Constant		123088.518	5895.414	20.879	0.000

Melard's algorithm was used for estimation.

In constructing the SARIMA-GRNN model, the simulation accuracy of the GRNN model was determined by using the smoothing factor δ.After exploring various smoothing factors from 0.1 to 1.0, we found when the smooth factor was 0.1, the hybrid model has lowest MSE, MAE and MAPE. So we selected 0.1 as an appropriate smoothing factor.

**Figure 3:**
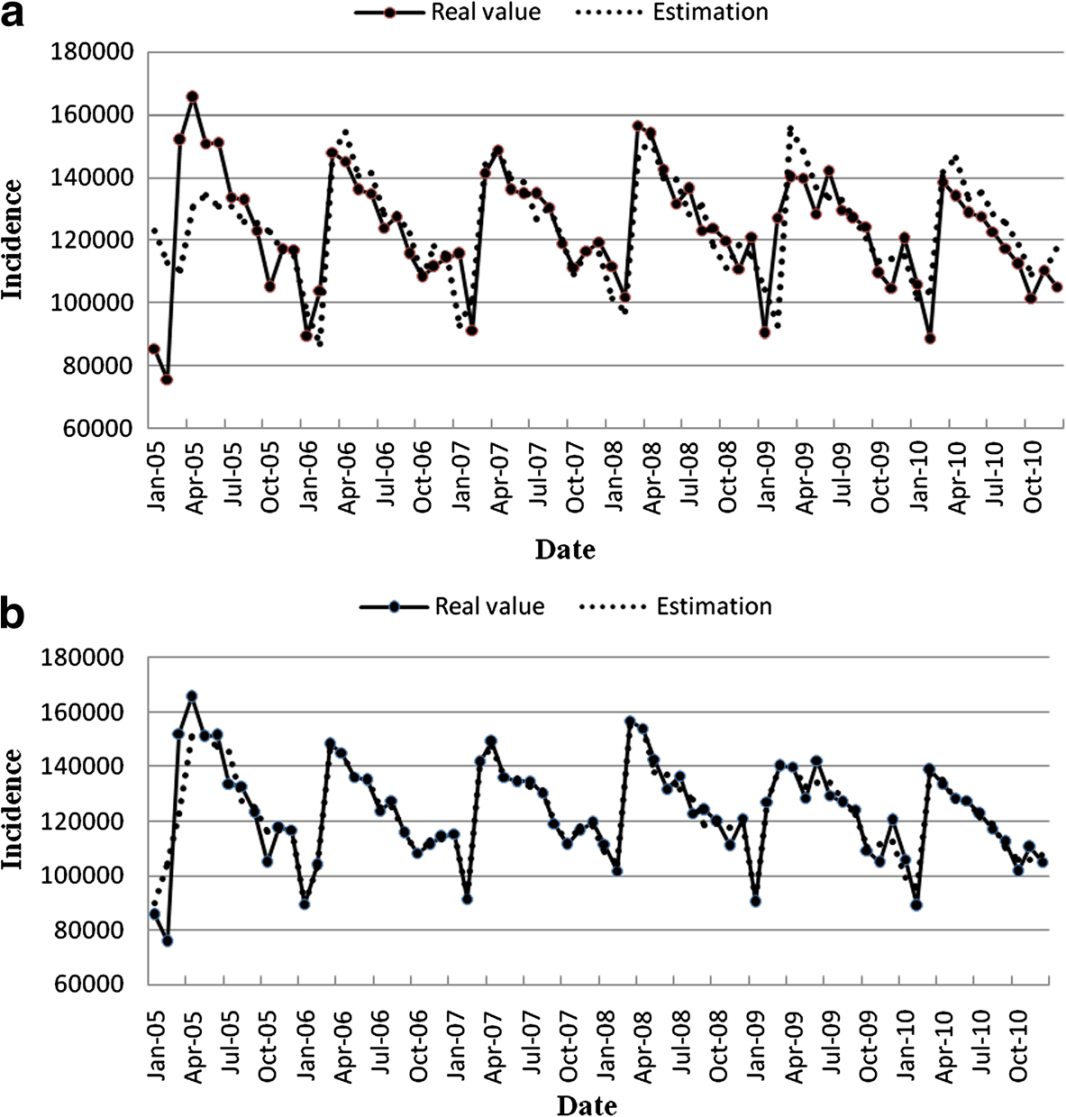
**Simulation effects of SARIMA model and SARIMA-GRNN model.** (**a**) Reported monthly incidence number of tuberculosis and its estimated value from SARIMA model; (**b**) Reported monthly incidence number of tuberculosis and its estimated value from SARIMA-GRNN model.

We compared SARIMA with SARIMA-GRNN in fitting goodness. The real incidence numbers and estimated incidence numbers of SARIMA model and SARIMA-GRNN model monthly are shown in Figure 3. MSE, MAE and MAPE of the combined model (38969150, 3406.593 and 0.030, respectively) were lower than those of the single SARIMA model (161835310, 8781.971 and 0.076, respectively).

We attempted to predict the number of monthly incident TB cases in 2011 using SARIMA and the hybrid models and compared them with the actual numbers (see Table 3). MSE, MAE and MAPE of the hybrid model were all lower than those of the single mode; and MAPE of the hybrid model was 3.7%.

**Table 3:** Reported and forecasted TB cases for 2011

**Date**	**Reported cases**	**Forecasted cases**	
		**SARIMA model**	**Hybrid model**
January	99617	97969	101914
February	98157	99085	104172
March	135848	144081	134218
April	129351	145607	134200
May	125129	135715	127165
June	119344	135855	127130
July	112647	129515	119442
August	115140	125768	110901
September	106925	119794	105334
October	100392	109934	108230
November	110662	112293	107170
December	104710	115860	105263
**Error**			
MSE		125120982.998	22749934.518
MAE		9737.576	4093.497
MAPE		0.084	0.037

From the model curve (Figure 3), we found that yearly TB incidence number in China showed a slightly decreasing trend. There was also a seasonal pattern in the number of new TB cases. The monthly incidence of TB was lower in January and February and higher from March to June.

## Discussion

We developed a SARIMA model and a SARIMA-GRNN hybrid model to predict monthly incidence of TB in China. Although both models could simulate the TB time series data well, the hybrid model, which takes both linear and nonlinear components into account, outperformed the SARIMA model. We believed that models combining ARIMA and artificial neural network contain more data characteristics than non-hybrid models and may thereby be better for forecasting.

Based upon the results of this study, we believe that there will be no obvious improvement in the high burden of TB in China in the near future. The results indicated that in the near future, the reported annual TB incidence numbers in China will decrease only slightly. Even though this study’s analysis was based only on reported cases of TB since time series data of real incidence of TB in China cannot be readily obtained, the web-based and case-based mandatory TB reporting system has been fully operational since 2005 and covers almost 100% of detected TB cases in China [16]. Hence, the reported incidence number should closely mirror the real incidence of TB. Moreover, the trends and epidemics from reported incidence numbers are very similar to those actual incidence and epidemic situation of TB. These findings reveal that the China’s progress in TB control has been very incremental and intensified interventions are urgently needed.

In this study we concluded that there is a seasonal variation in TB incidence that showed periodicity in China. The monthly incidence number demonstrated a trough in January and February and was higher from March to June. This seasonal trend of TB incidence in China differed from that of many other nearby regions. For instance, in Hong Kong, the peak season of TB incidence is summer only [17]. In northern India the peak time season of TB incidence occurred between April to June and in October and December; although the TB incidence was lower in other months, there was no obvious seasonal trends [18]. One plausible explanation of these seasonal trends in China may be that the annual Spring Festival, the most important traditional festival of the year, usually falls in mid-February. During the entire month of February, there are huge population movements throughout China by various transportation modes. In view the TB incubation period, we suggest that the peak time of TB incidence may be partly attributed to the large population flows and the poor ventilation of public transportation during the Spring Festival period. The Spring Festival period may be the peak time of TB transmission. Hence, measures to prevent TB transmission within public transportation during the Spring Festival may play an important role in decreasing incidence number particularly from March to June. Other possible mechanisms for the seasonal variations in TB incidence in China need to be further studied.

There are a number of limitations in present study as follow. Firstly, climate–related data and data related to population movements were not included in the model fitting because of limitations in data availability. Secondly, China is a vast country and with a wide variation in climate, so seasonality of TB in the various geographic regions may differ. Due to lack of available data, seasonality at smaller area levels was not been analyzed. Lastly, the models were derived using data only from 2005 through 2010 and tested against only one year of data. Hence, these findings should be interpreted with caution and may be re-examined with additional time series data.

## Conclusions

Limitations inherent in non-hybrid forecasting models can be compensated by developing applying hybrid model that combines two or more models. The resulting hybrid model may thereby be more effective than a single model in producing reliable forecasts of TB. Since the models suggest that the TB epidemic in China will not decrease markedly in the coming years, there is a need to implement greater TB control measures in China. The seasonality of the TB incidence suggested by the models also indicate the need for interventions focused on reducing infectious disease transmission on public transportation during the Spring Festival period.

## Abbreviations

TB: Tuberculosis; SARIMA: Seasonal autoregressive integrated moving average; GRNN: Generalized regression neural network; MSE: Mean square error; MAE: Mean absolute error; MAPE: Mean absolute percentage error; MOH: Ministry of Health of the People’s Republic of China.

## Competing interests

All authors declared no competing interests.

## Authors’ contributions

ZL and SC conceived the idea and prepared a draft protocol. SC and JL collected relevant data. SC analyzed data and prepared the manuscript. FW, WT, JL, JK and ZL helped results interpretation and critically commented on and revised the manuscript. All authors read and approved the final manuscript.

## Authors’ information

Shiyi Cao: Doctoral candidate in Tongji Medical College, Huazhong University of Science and Technology

Feng Wang: Research Assistant Professor in School of Public Health and Primary Care, the Chinese University of Hong Kong

Wilson Tam: Research Assistant Professor in School of Public Health and Primary Care, the Chinese University of Hong Kong

Lap Ah Tse, Assistant Professor in School of Public Health and Primary Care, the Chinese University of Hong Kong

Jean Hee Kim, Assistant Professor in School of Public Health and Primary Care, the Chinese University of Hong Kong

Junan Liu: Associate professor in Tongji Medical College, Huazhong University of Science and Technology

Zuxun Lu: director and professor in Tongji Medical College, Huazhong University of Science and Technology

## Pre-publication history

The pre-publication history for this paper can be accessed here:

http://www.biomedcentral.com/1472-6947/13/56/prepub
